# Medication and health-related outcomes by prescription lengths: A systematic review

**DOI:** 10.1016/j.rcsop.2026.100862

**Published:** 2026-10-14

**Authors:** Su Myat Thin, Parnnaphat Luksameesate, Chanthawat Patikorn, Suthira Taychakhoonavudh

**Affiliations:** Department of Social and Administrative Pharmacy, Faculty of Pharmaceutical Sciences, Chulalongkorn University, Bangkok, Thailand

**Keywords:** Prescription length, Medication adherence, Medication waste, Costs

## Abstract

**Background:**

Prescription length policies (such as 30, 60, or 90 days) influence medication adherence, healthcare access, costs, and patient safety. However, their overall impact remains unclear because of heterogeneous evidence.

**Objectives:**

This systematic review evaluated the effects of prescription length on clinical, economic, and healthcare system outcomes and identified methodological considerations for future research and policy.

**Methods:**

PubMed, Scopus, and ScienceDirect were searched for studies published from 2000 to July 1, 2026. Studies comparing different prescription lengths were included, while those unavailable in full text were excluded. Methodological quality was assessed using design-specific critical appraisal tools. Due to substantial heterogeneity in study designs, populations, prescription durations, and outcome measures, findings were synthesized narratively.

**Results:**

Twenty-three studies were included. Longer prescription durations were generally associated with improved medication adherence and increased medication waste. Evidence for economic, clinical and healthcare utilization outcomes was more limited and heterogeneous. Among studies of chronic disease management, longer prescription durations were generally associated with lower out-of-pocket costs, transportation cost and patient time cost, and reduced healthcare encounters, although findings for total economic outcomes varied according to the analytical perspective adopted. For acute conditions with opioid use, shorter prescription durations were associated with reduced adverse outcomes, including overdose risk and persistent opioid use. Methodological heterogeneity in study design, outcome definitions, and analytical perspectives contributed to differences in findings across studies.

**Conclusion:**

Longer prescription lengths were consistently associated with improved medication adherence but also increased medication waste. Evidence for clinical, economic, and healthcare system outcomes remains limited and heterogeneous and should be interpreted according to medication type and clinical context. Thus, a single prescription duration is unlikely to be appropriate across all settings. Healthcare providers and policymakers should consider medication type, patient needs, treatment stability, and healthcare system context when determining prescription duration. Further well-designed studies using transparent and comparable outcome definitions are needed to strengthen the evidence base for prescription-length decision-making.

## Highlights

• Prescription lengths affect clinical, economic, and healthcare outcomes.
• Longer prescriptions improved adherence and reduced healthcare costs.
• Longer prescription lengths increased medication waste in chronic disease medication settings.
• Optimal prescription length depends on medication and clinical context.

## 1 Introduction

Prescription length policy, governing the maximum days' supply of a medication that can be dispensed at one time, is a critical component of healthcare management.1., 2., 3., 4. These policies typically vary between 30, 60, and 90 days, and the debate over the ‘optimal’ duration has intensified as healthcare systems seek to balance improvements in medication adherence and clinical outcomes against risks including costs, wastage, and drug misuse.1., 2., 3., 4. Despite extensive research on prescription length, important uncertainty remains regarding how prescription duration should be determined in different clinical and healthcare settings.5., 6., 7. Decisions about prescription duration are influenced by multiple factors, including medication characteristics, treatment goals, patient needs, healthcare delivery models, and resource availability. Consequently, evidence generated in one therapeutic area or healthcare setting may not be directly applicable to another, making it difficult for clinicians, researchers, and policymakers to interpret the overall evidence and apply it appropriately in practice.

Existing studies have reported inconsistent findings regarding the impact of prescription length on patients' clinical and economic outcomes, as well as other aspects of the health system. Some studies reported that longer prescription lengths are associated with improved medication adherence and cost savings,2., 4., 8., 9., 10., 11., 12. while others highlighted increased medication wastage and drug misuse as potential harm.9., 13., 14., 15., 16., 17. Previous systematic reviews primarily focused on medication adherence and, also on medication waste. This review extends previous research by synthesizing evidence across clinical, economic, and healthcare system outcomes, while examining how methodological variation affects the comparability and interpretation of findings across studies.

Given these contradictory findings, a comprehensive synthesis of the evidence is necessary to inform best practices. Therefore, this systematic review aimed to comprehensively evaluate the outcomes associated with varying prescription lengths across multiple outcome domains, while also examining the methodological approaches, including study designs, outcome definitions, and measurement methods, used to assess these outcomes across studies. By integrating evidence across diverse study designs, medications, and healthcare settings, this review seeks to provide a broader evidence base to support context-specific decision-making regarding prescription duration.

## 2 Method

### 2.1 Data sources and searches

The PRISMA (Preferred Reporting Items for Systematic Reviews and Meta-Analysis) 2020 statement was used to report this systematic review.^[18]^ The keywords used in data searching were (prescription length) OR (prescription duration) OR (medication duration) OR (prescription period) OR (extended prescription) OR (dispensing duration) OR (prescription size) OR (prescription interval) OR (prescription pattern). Three databases (PubMed, Scopus, and ScienceDirect) were used for data search because together they provided broad coverage of biomedical, pharmaceutical, and multidisciplinary literature relevant to prescription-length research. According to the data search guidelines for these databases, the search terms used were different (Appendix A). The search was limited to studies published from 2000 onwards because prescription dispensing practices, healthcare delivery systems, and medication management strategies have evolved substantially over the past two decades. Restricting the search to this period was intended to ensure that the evidence synthesized is relevant to contemporary clinical practice and healthcare systems. The search period was from 2000 to 1st July 2026. EndNote 20 was used to manage references and organize the articles included in this systematic review.

### 2.2 Study selection and quality assessment

Inclusion criteria for studies were the following: (1) primary research that reported outcomes or consequences affected by changing into longer or shorter prescription lengths, (2) in the English language, and (3) retrieved with full text. Studies that described prescription length but did not report any outcomes of changes in prescription length were excluded. Initial screening of the titles and abstracts was performed by one researcher (S.M.T.) to identify potential studies according to the inclusion criteria. Other researchers (C.P. and P.L.) independently checked them and any disagreements or uncertainties were resolved through discussion until consensus was reached. The methodological quality of the included studies was assessed using design-specific appraisal tools appropriate to each study design (in Appendix B).^[19]^ The Newcastle–Ottawa Scale (NOS)^[20]^ was used for non-randomized studies; the JBI Critical Appraisal Checklist^[21]^ was for quasi-experimental studies; the ECOBIAS checklist^[22]^ was used to assess the risk of bias of economic evaluation studies; the Mixed Methods Appraisal Tool (MMAT) 2018 ^[23]^ was for mixed-method studies; the JBI Critical Appraisal Checklist for Analytical Cross-Sectional Studies^[24]^ was for cross-sectional studies; and the JBI Critical Appraisal Checklist for Qualitative Studies^[25]^ was for a qualitative study. Two reviewers (S.M.T. and C.P.) independently conducted quality assessments, with disagreements resolved by consensus or a third reviewer (S.T.). Studies that did not meet the predefined quality criteria were excluded to ensure that the review synthesized evidence from studies with adequate methodological rigor and reporting quality.

### 2.3 Data extraction

The data extracted were about (1) the duration or length of medication prescription and (2) outcomes affected by changes in prescription length. Moreover, the authors, study location, study population, sample size, participants' age, studied diseases, and medication used in each study were also extracted. The data extraction was performed by one researcher (S.M.T.), and the other two researchers validated the extracted data (C.P. and P.L.).

### 2.4 Data synthesis and analysis

All data were descriptively summarized and reported with a narrative synthesis due to the anticipated heterogeneity in study designs, populations, medication lengths, and outcome measures (e.g., different definitions of “wastage” or adherence metrics). Identified countries were categorized based on the World Bank's income-level classification.^[26]^ Findings were grouped by outcome category, such as medication adherence, waste, cost, and drug abuse. Results were tabulated to compare the direction of effects for each outcome measure by prescription length. Cost data were extracted and reported in the original currency and units as presented in the included studies, without currency conversions or cost standardizations. Two researchers (S.M.T. and S.T.) performed the data analysis. Microsoft Excel (Office 365) was used for data management. There was a group discussion with all authors to discuss the discrepancy. This systematic review was not prospectively registered.

## 3 Results

A total of 1709 records were initially identified across the databases and sources of references and citation searches (Fig. 1). The list of excluded full-text studies, with reasons for exclusion, is provided in Appendix C. None of the eligible studies were excluded due to a high risk of bias. After excluding articles that did not meet the inclusion criteria, 23 studies were included in the final systematic review (Fig. 1).1., 2., 3., 4., 8., 9., 10., 11., 12., 13., 14., 15., 16., 17., 27., 28., 29., 30., 31., 32., 33., 34., 35., 36., 37., 38., 39., 40., 41., 42.

**Fig. 1:**
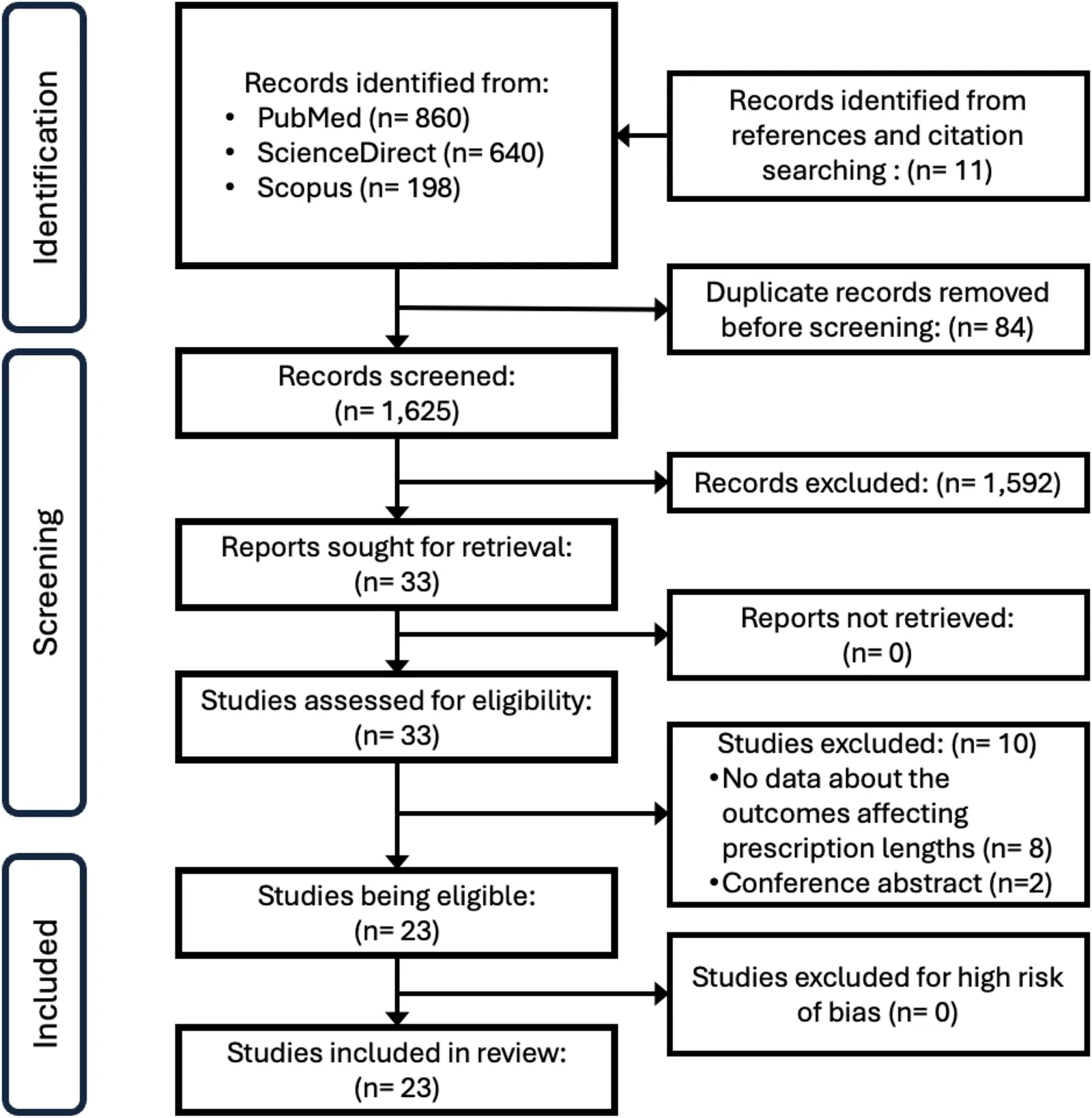
Study selection flow chart.

### 3.1 Characteristics of the included studies

Of the included studies, 17 (74%) were conducted in high-income countries, including 13 in the United States,1., 2., 3., 8., 9., 10., 13., 15., 27., 28., 29., 30., 38. 2 in the United Kingdom,16., 31. one each in Taiwan,^[37]^ and Canada^[39]^ (in Table S1). Five studies (22%) were from upper-middle-income countries, including South Africa,11., 36. Thailand,^[4]^ China,^[32]^ and Malaysia.^[40]^ One study (4.3%) was conducted in Haiti, a lower-middle-income country.^[35]^

Sample sizes varied considerably across studies, ranging from small cohorts of 30 participants to large administrative datasets of over two million individuals. The most common study design was retrospective cohort (14 studies, 61%),2., 3., 9., 10., 13., 16., 28., 30., 32., 35., 37., 38., 39., 40. followed by quasi-experimental (4 studies, 17.4%).1., 4., 27., 29. Others were two simulation studies (8.7%),11., 15. one cross-sectional study (4.3%),^[8]^ one qualitative study (4.3%)^[31]^ and one mixed-method study (4.3%).^[36]^ Retrospective cohort studies were most commonly used and relied on large administrative or claims datasets to evaluate outcomes such as medication adherence, medication waste, adverse medication use, and healthcare utilization.2., 3., 9., 10., 13., 16., 28., 30., 32., 35., 37., 38., 39., 40. Quasi-experimental studies1., 4., 27., 29. evaluated the effects of policy-driven changes in prescription length, whereas simulation studies projected long-term clinical and economic outcomes resulting from the changes in prescription length.11., 15. Cross-sectional and mixed-method studies provided evidence on prescription length comparisons at a single time point and explored patient and provider perspectives related to prescription duration.8., 31., 36.

Studies addressed a range of clinical conditions and compared various prescription durations. Seventeen studies (74%) focused on patients with chronic non-communicable diseases (NCDs), including hypertension, diabetes, dyslipidemia, cancer, psychosis, and other long-term conditions.1., 2., 3., 4., 8., 9., 10., 11., 13., 15., 16., 27., 28., 29., 30., 31., 32. The most frequent comparison was 30-day versus 90-day supplies which was compared in 6 studies (26.1%).2., 4., 9., 13., 27., 28. Other studies evaluated <60 days versus >60 days,^[16]^ <90 days versus >90 days,^[3]^ and 30 days versus 60 days.8., 11., 30. Three studies assessed outcomes related to changes in prescription length from 100 days to 34-days.1., 15., 29. Two studies focused on HIV patients compared long prescription lengths of 1–2 months with 3-month or 5-month durations.35., 36. Three studies investigated opioid-related pain management evaluated shorter prescription durations, such as <3 days, 3–7 days, >7 days, or > 15 days.38., 39., 40. One study on smoking cessation examined durations of 1 week, 2 weeks, and > 3 weeks.^[37]^

Outcomes were reported across eight categories: medication adherence (*n* = 10),1., 4., 8., 9., 10., 27., 29., 30., 32., 36. medication waste (*n* = 4),2., 9., 13., 16. economic outcomes (*n* = 7),1., 2., 9., 11., 15., 16., 28. adverse medication use (*n* = 3),38., 39., 40. clinical outcomes (*n* = 3),11., 30., 37. healthcare utilization (*n* = 2),3., 36. retention in care (*n* = 1),^[35]^ and patient satisfaction (*n* = 2)31., 36. (Table 1). Given the heterogeneity in outcome definitions and measurement approaches, findings were synthesized narratively according to outcome category.

**Table 1:**
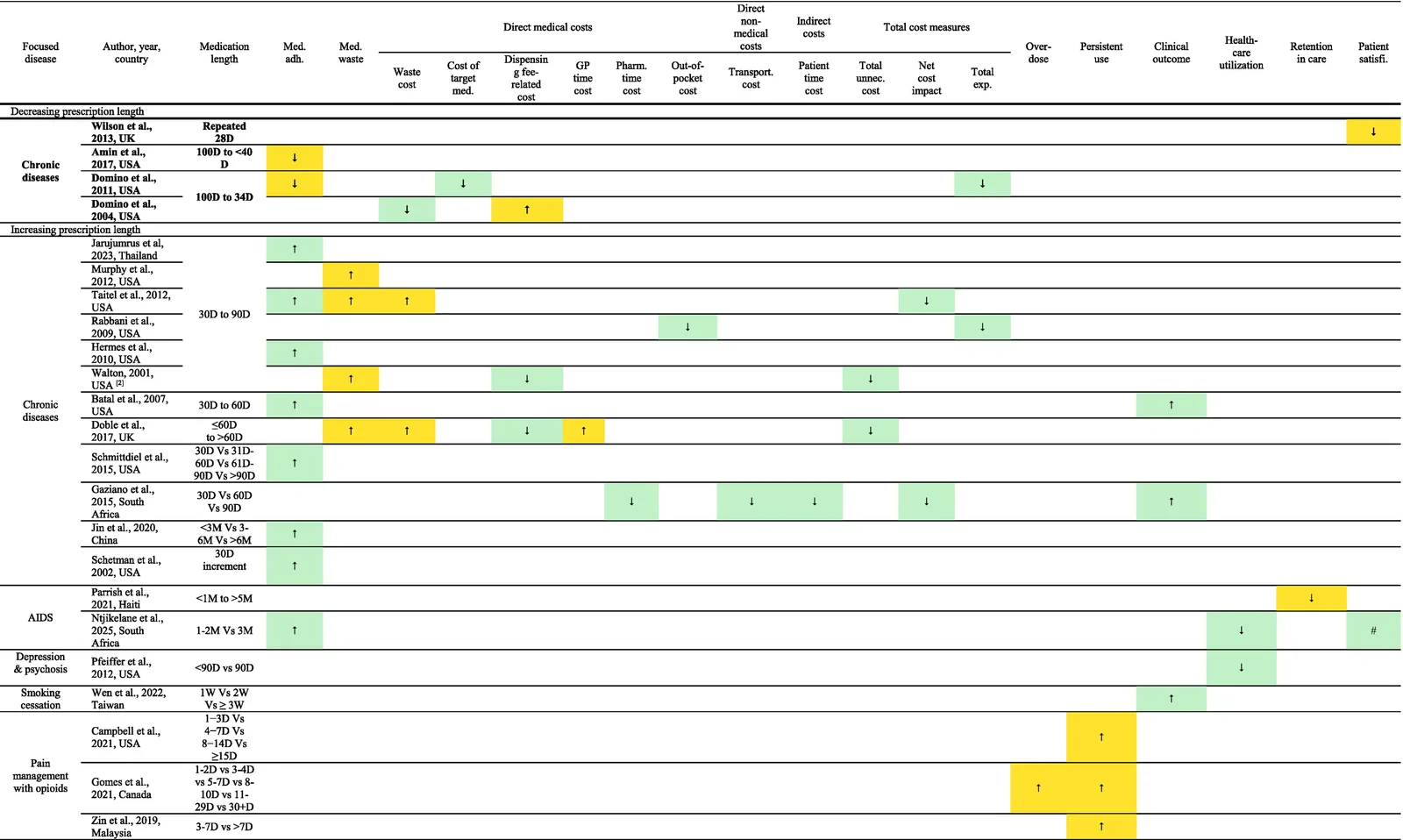
Summary of reported outcomes across different prescription lengths in the included studies.

Note: ↑ = increased in an outcome while changing prescription lengths, ↓ = decreased in an outcome while changing prescription lengths, Med. = medication, adh. = adherence, exp. = expenditure, Pharm. = pharmacist, unnec. = unnecessary, Transport. = transportation, satisfy. = satisfaction, D = days, W = weeks, M = months, the studies with **bold** letter conducted the outcomes from changing longer duration (100 days) to shorter duration (<40 days or 34 days). Green color = beneficial impact on outcomes while changing prescription lengths, Yellow color = harmful impact on outcomes while changing prescription lengths,

The methodological quality of the included studies was assessed using design-specific critical appraisal tools (Appendix B). Overall, the methodological quality of the included studies was high. All 14 retrospective cohort studies were rated as high quality.2., 3., 9., 10., 13., 16., 28., 30., 32., 35., 37., 38., 39., 40. All four quasi-experimental studies were judged to be of high quality, meeting most JBI appraisal criteria.1., 4., 27., 29. Of the two simulation studies, one demonstrated high reporting quality,^[11]^ whereas the other was rated as moderate due to incomplete reporting of several CHEERS 2022 items.^[15]^ The mixed-methods,^[36]^ analytical cross-sectional,^[8]^ and qualitative studies^[31]^ were all considered to be of high quality, with only minor limitations, such as insufficient reporting of researcher reflexivity in the qualitative study. Overall, the quality assessment indicated that the included evidence was generally robust and suitable for the inclusion in this systematic review.

### 3.2 Evidence synthesis: outcomes associated with varying prescription lengths

#### 3.2.1 Medication adherence

Medication adherence was the most frequently reported outcome, examined quantitatively in 10 studies across multiple study designs and chronic disease populations.1., 4., 8., 9., 10., 27., 29., 30., 32., 36. Overall, these studies found that longer prescription durations were associated with higher medication adherence, with consistent findings across retrospective cohort, quasi-experimental, and cross-sectional designs (Table 2).

**Table 2:**
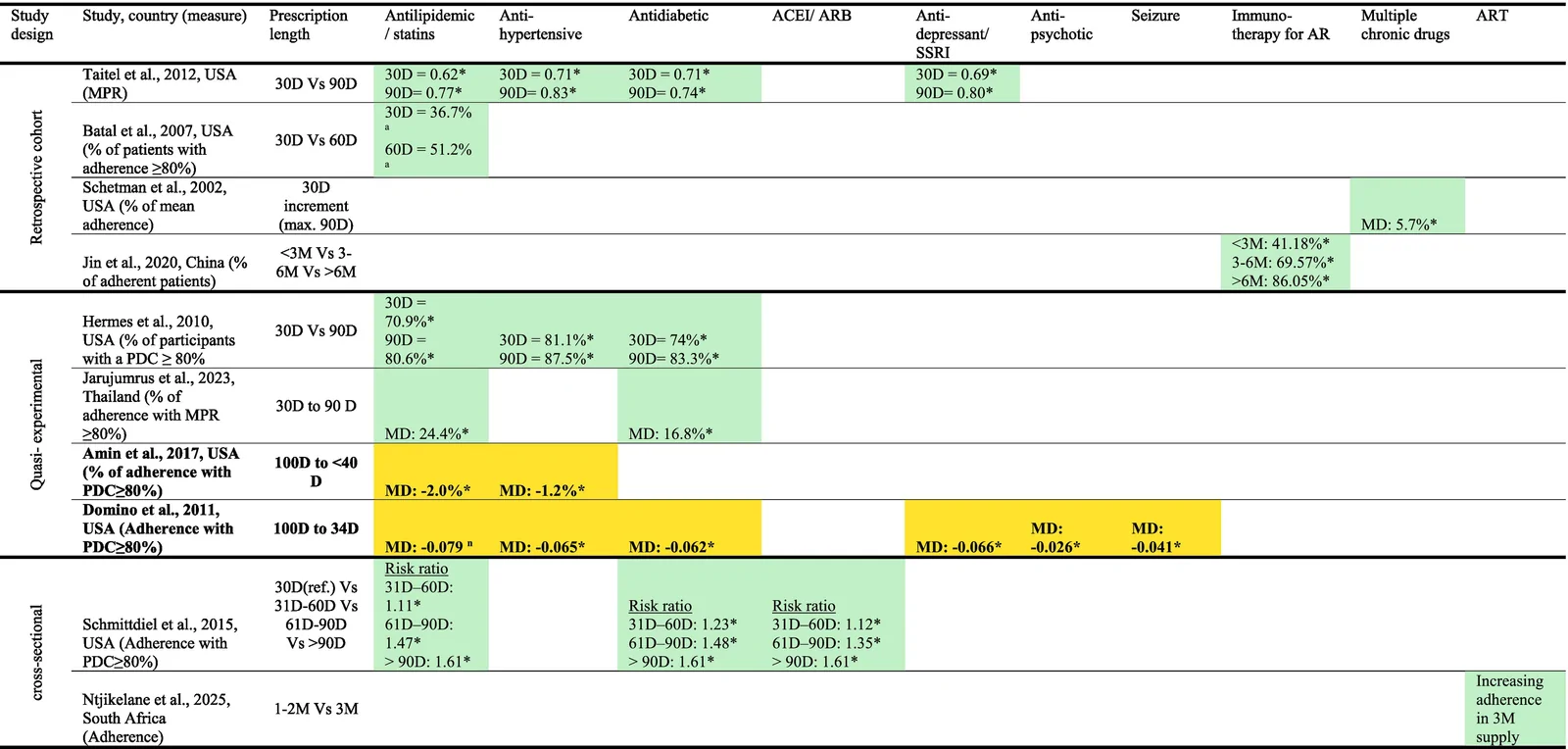
Medication adherence outcome.

Note: ACEI = angiotensin-converting enzyme inhibitor, ARB = angiotensin II receptor blocker, SSRI = selective serotonin reuptake inhibitor, AR = allergic rhinitis, ART = antiretroviral therapy, MPR = medication possession ratio, PDC = proportion of days covered, D = days, M = months, * = significant, ^a^ = no data about significance of the result, ^n^ = non-significant, MD = Mean Difference, Green color = beneficial impact on outcomes while changing prescription lengths, Yellow color = harmful impact on outcomes while changing prescription lengths, the studies with bold letter conducted the outcomes from changing longer duration (100 days) to shorter duration (<40 days or 34 days).

In the retrospective cohort study, adherence measured by medication possession ratio (MPR) was higher with 90-day prescriptions compared with 30-day supplies, including antilipidemic (0.77 vs 0.62), antihypertensive (0.83 vs 0.71), antidiabetic (0.74 vs 0.71), and antidepressant medications (0.80 vs 0.69).^[9]^ Similarly, the proportion of adherent patients increased with longer durations, such as 51.2% for 60-day versus 36.7% for 30-day prescriptions.^[30]^ In quasi-experimental studies, extending prescription length from 30 to 90 days improved adherence (e.g., increases of 16.8%–24.4%),^[4]^ whereas shortening prescription duration (from 100 days to 34–40 days) was significantly associated with reductions in adherence across multiple drug classes.1., 29. A cross-sectional study also supported a dose–response relationship, with adherence increasing progressively as prescription duration extended, with risk ratios up to 1.61 for >90 days compared with 30 days.^[8]^ Complementing the quantitative evidence, a qualitative study among healthcare providers in South Africa reported that a 3-month ART supply enhanced medication adherence compared with 1 to 2-month supplies, though this finding reflected provider perceptions rather than directly measured patient adherence.^[36]^

#### 3.2.2 Medication waste

Four studies reported medication waste outcomes, and the overall direction of evidence was consistent; longer prescription lengths were associated with greater medication waste across most drug classes and measurement approaches (Table 3).2., 9., 13., 16. Nevertheless, one study highlighted that this pattern was not uniform across all drug classes, as 90-day supplies of antidepressants produced lower day-supply wastage than 30-day supplies; however, the difference did not reach statistical significance.^[13]^ It indicated that the impact of prescription length on medication waste may vary across drug classes.

**Table 3:**
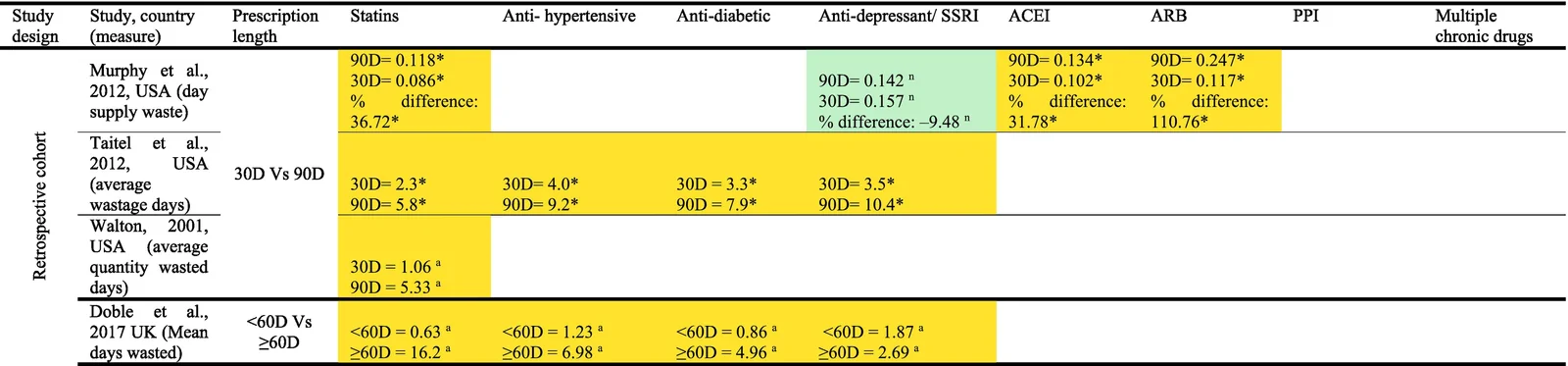
Medication waste outcome.

Note: D = days, * = significant, a = no data about significance of the result, n = non-significant, ACEI = angiotensin-converting enzyme inhibitor, ARB = angiotensin II receptor blocker, SSRI = selective serotonin reuptake inhibitor, PPI = proton pump inhibitors, Green color = beneficial impact on outcomes while changing prescription lengths, Yellow color = harmful impact on outcomes while changing prescription lengths,

#### 3.2.3 Economic outcomes

Cost outcomes in included studies were grouped into two main categories: component cost measures which are specific cost elements related to prescription practices, including direct medical costs, direct non-medical costs, indirect costs, and total cost measures (Table 4).

**Table 4:**
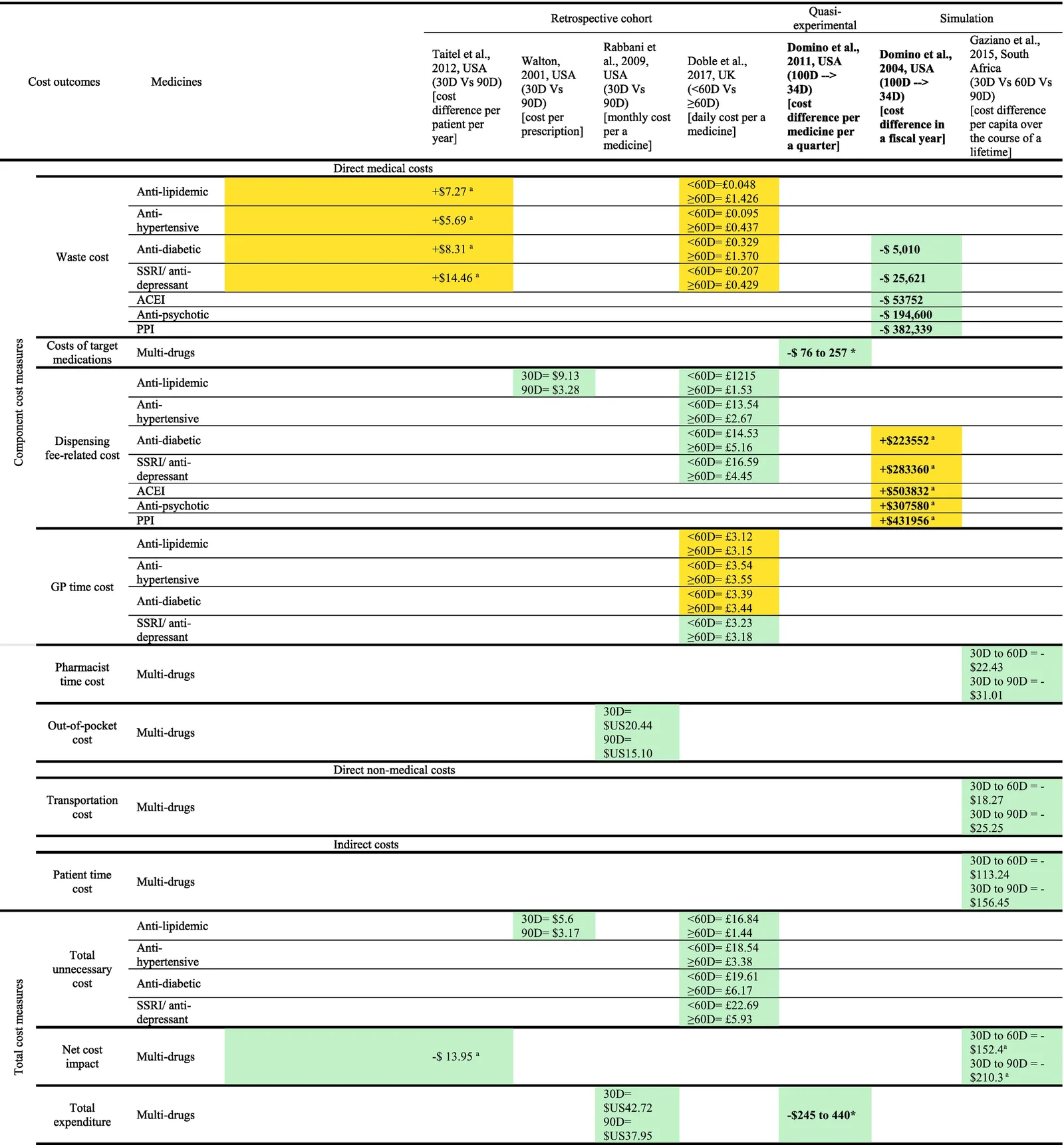
Economic outcomes.

Note: ACEI = angiotensin-converting enzyme inhibitor, ARB = angiotensin II receptor blocker, SSRI = selective serotonin reuptake inhibitor, PPI = proton pump inhibitors, * = significant, a = no data about significance of the result, D = days, Green color = beneficial impact on outcomes while changing prescription lengths, Yellow color = harmful impact on outcomes while changing prescription lengths, the studies with bold letter conducted the outcomes from changing longer duration (100 days) to shorter duration (<40 days or 34 days).

##### 3.2.3.1 Component cost measures

Direct medical cost measures associated with prescription length identified in the included studies included costs of target medication, waste costs, dispensing fee-related costs, general practitioner (GP) time costs, and pharmacist time costs.1., 2., 9., 11., 15., 16. Waste costs were generally higher in longer prescription durations in two retrospective cohort studies comparing 30-day vs 90-day and < 60-day vs ≥60-day supplies.9., 16. A simulation study similarly demonstrated substantial reductions in waste costs with shorter supply (34 days) following a policy change from 100-day to 34-day dispensing.^[15]^ Shortening prescription length from 100 to 34 days reduced quarterly costs of target medications across six chronic medication classes. The reduction varied by medication class, ranging from US$76 to US$257 immediately after implementation (Table 4).^[1]^ GP time costs differed only marginally by prescription duration, with differences of approximately £0.01 to £0.05 per prescription.^[16]^ Four medication classes showed slightly lower GP time costs with shorter prescription durations, whereas antidepressants showed a slightly lower GP time cost with longer prescription duration. In contrast, dispensing fee-related costs were consistently higher with shorter prescriptions, due to more frequent dispensing.2., 15., 16. Pharmacist time costs were also slightly higher with shorter durations, due to increased dispensing workload.^[11]^ Shorter prescription durations resulted in higher out-of-pocket costs (30 days, $20.44, 90 days: $15.1), indicating a greater financial burden on patients comparted with longer prescription durations.^[28]^

Transportation costs were the only direct non-medical cost measure identified in the included studies. Extending prescription duration from 30 days to 60 days and from 30 days to 90 days resulted in transportation cost savings of $18.27 and $25.25, respectively, indicating increased patient burden from more frequent visits under shorter prescriptions.^[11]^ Indirect costs, including patient time costs, were lower in longer prescription lengths, with a reduction ranging from $113.24 to $156.45, reflecting time lost from repeated healthcare visits under shorter prescriptions.^[11]^

##### 3.2.3.2 Total cost measures

Total cost measures refer to overall cost outcomes reported in the included studies that capture the aggregate economic impact of different prescription lengths. These measures included total unnecessary costs, net cost impacts, and cost savings. Total unnecessary cost (TUC) showed mixed patterns, which were a combination of waste cost and dispensing cost with or without costs of prescriber times (detailed definitions in Table S2). Two retrospective studies indicated lower TUC with longer prescriptions.2., 16. In one study, TUC was $5.6 for 30-day prescriptions and $3.17 for 90-day prescriptions (Table 4).^[2]^ In another study, TUC for prescription length < 60 days ranged from £ 1.44 to £ 6.17, while TUC for ≥60-day durations ranged from £ 16.84 to £ 22.69.^13^

The net cost impact was examined as cost savings in two studies and varied depending on the definitions (shown in Table S2). In this net cost impact, cost savings were generally observed with longer prescription durations. A retrospective study reported savings of $13.95 when prescription duration is longer.^[9]^ A simulation study estimated total annual per-patient cost savings of $152.4 and $210.3 for 60-day and 90-day prescription durations, respectively, compared with 30-day prescriptions.^[11]^ Total expenditure findings varied by perspective, which was mentioned in detail in Table S2.1., 28. A medication-focused study showed lower total healthcare expenditure with longer prescriptions ($42.72 vs $37.95).^[28]^ In contrast, a system-level study reported lower total healthcare expenditures with shorter prescription durations, with savings ranging from US$245 to US$440 per person per quarter across medication classes.^[1]^

#### 3.2.4 Adverse medication outcomes

In three studies, data on adverse medication outcomes (Table S3) demonstrated a clear association between longer prescription durations of opioids and increased risks of overdose and persistent use.38., 39., 40. Opioid overdose risk increased significantly with longer durations, from an HR (Hazard Ratio) of 1.27 in the 5–7 days prescription length group to an HR of 1.80 in the 11–29 days prescription length group.^[39]^ Persistent opioid use demonstrated a strong duration-dependent relationship, with risks escalating significantly for prescriptions exceeding 7 days.38., 39., 40. Adjusted odds ratios (AOR) for persistent use ranged from 1.22 for 8–14 days^[38]^ to as high as 16.47 for prescriptions exceeding 7 days.^[40]^ Furthermore, the risk peaked at an AOR of 8.02 for durations of 30 days or longer,^[39]^ while the use of long-acting opioids further compounded the risk (AOR 7.88).^[38]^

#### 3.2.5 Clinical outcomes

Three studies found that longer prescription durations for chronic and preventive therapies generally improve clinical outcomes (Table S4).11., 30., 37. Specifically, 60-day statin supplies achieved lower cholesterol levels than 30-day supplies. In a simulation study, it was projected that 2553 additional lifesaving events occurred with 90-day durations, while 30-day durations had 1694 lifesaving events.11., 30. Similarly, longer varenicline prescription length significantly increased the odds of successful smoking cessation (OR: 1.19).^[37]^

#### 3.2.6 Healthcare utilization

In this review, healthcare utilization refers to the use of healthcare services, including patient clinic visits and clinical encounters, reflecting the frequency and occurrence of patient interactions with healthcare providers.3., 36. Two studies found that healthcare utilization, particularly follow-up visits, was significantly reduced in patients with extended prescription durations (Table S5).3., 36. For patients prescribed with antidepressants, 90-day supplies nearly halved re-consultation rates (13.7% vs. 24.9%).^[3]^ In an antiretroviral therapy (ART) study, 87% of healthcare providers reported that 3-month ART supplies reduced clinic visits.^[36]^

#### 3.2.7 Retention in care

Retention in care was reported in one study of ART, where it was defined as timely ART refill pickup, specifically patients collecting their ART refill within 30 days of the scheduled pickup date at 12 months (Table S2). Compared with 30-day prescriptions, 110-day prescriptions were associated with a smaller gain in retention, suggesting that longer prescription lengths may reduce retention in care (Table S5).^[35]^

#### 3.2.8 Patient satisfaction

Longer prescription durations improved patients' convenience as well as patients' satisfaction, as observed in two studies.31., 36. It was found that 76% of providers considered 3-month ART supplies more convenient for patients than 1–2-month supplies.^[36]^ Additionally, a qualitative study showed that patients reported lower satisfaction with 28- or 30-day durations for multiple medications compared with durations of more than 30 days (Table S5).^[31]^

## 4 Discussion

This systematic review provides a comprehensive synthesis of the global evidence on the impact of varying prescription lengths across multiple clinical, economic, and healthcare outcomes. The findings consistently demonstrate that longer prescription durations were generally associated with improved medication adherence, while also increasing medication waste, although the magnitude of adherence improvement varied across medication classes. These two outcomes were the most frequently evaluated and showed the most consistent evidence across the included studies. In contrast, evidence for economic outcomes, healthcare utilization, clinical outcomes, adverse medication outcomes, retention in care, and patient satisfaction remained inconclusive. The included studies represented diverse therapeutic areas, including cardiovascular diseases, diabetes, mental health conditions, HIV, smoking cessation, and opioid prescribing, and the effects of prescription length were not uniform across these clinical contexts. It should be noted that most included studies focused on chronic disease management. Studies of opioid prescribing for acute conditions demonstrated a different pattern, where shorter initial prescription durations were consistently associated with lower risks of persistent opioid use and overdose. Similarly, medication-specific findings reported throughout this review demonstrated that the magnitude and direction of the observed effects varied according to the medication and clinical context. These findings suggest that a single prescription length is unlikely to be appropriate across all clinical settings and medication classes.

Our findings build on those in previous review studies,5., 6., 7. in which longer prescription durations for long-term medications were found to improve medication adherence while increasing medication waste. Our review extends these findings by demonstrating that the effects of prescription length differ by medication type and clinical context rather than assuming a uniform effect across all therapies. Unlike previous reviews, we also synthesized medication- and disease-specific findings across multiple outcome domains, allowing differences between chronic therapies, preventive therapies, antiretroviral therapy, mental health medications, and opioid prescribing to be interpreted within their respective clinical contexts. In addition, our review provides a framework for interpreting the heterogeneous economic evidence by distinguishing between component cost measures and total cost measures. This distinction highlights how differences in economic perspective and the cost components included can lead to different conclusions regarding the economic impact of prescription length.

The association between longer prescription durations and improved medication adherence may be explained by the reduced burden of obtaining medications through less frequent prescription refills and pharmacy visits.7., 36. Medication adherence is influenced by multiple patient-, therapy-, socioeconomic-, and healthcare system-related factors, with treatment burden and healthcare access recognized as important determinants.6., 7. Extending prescription duration may therefore improve adherence by reducing logistical barriers associated with obtaining medications and simplifying long-term medication management.

Longer prescription durations were consistently associated with increased medication waste across the included studies. This finding may be explained by the larger quantities of medication dispensed at each refill, which increase the likelihood that unused medicines remain when therapy is modified or discontinued before the dispensed supply is exhausted. Among the included studies, Doble et al. quantified medication waste arising from early repeat prescriptions and treatment changes, including dosage adjustments and medication switching.^[16]^ These findings suggest that the risk of medication waste depends not only on prescription duration but also on the likelihood of treatment modification during the dispensing interval. Longer prescription durations are likely to be most appropriate for patients with stable treatment regimens, while patients with anticipated treatment changes may benefit from shorter dispensing intervals to minimize unnecessary medication waste.

Evidence regarding economic outcomes was more heterogeneous than that for medication adherence and medication waste. This heterogeneity largely reflected differences in the economic perspective adopted and the cost components evaluated across studies rather than conflicting evidence. Studies examining individual cost components demonstrated expected trade-offs, with longer prescription durations generally increasing medication waste costs while reducing dispensing fees, prescriber time, patient out-of-pocket costs, and other transaction-related costs. In contrast, findings from studies reporting total cost measures varied according to the economic perspective adopted and the cost components included in the analysis. These findings highlight the importance of distinguishing between component cost measures and total cost measures when interpreting the economic impact of prescription length, as differences in analytical perspective and included cost components may lead to different conclusions. Future economic evaluations of prescription-length policies should clearly define the analytical perspective and justify the cost components included. Where appropriate, adopting a societal perspective may provide a more comprehensive assessment by capturing opportunity costs and spillover effects across all stakeholders affected by the policy, including patients, healthcare providers, payers, and community pharmacies. Such evaluations would better inform policy decisions and identify whether complementary measures are needed to mitigate unintended consequences of prescription policy experienced by specific stakeholder groups.

Healthcare utilization, clinical outcomes, retention in care, and patient satisfaction were examined in relatively few studies, with substantial variation in study populations, outcome definitions, and follow-up periods, making it difficult to draw firm conclusions regarding the impact of prescription length. In addition, these outcomes are influenced by multiple clinical, behavioral, and healthcare system factors beyond prescription duration, making it difficult to isolate the independent effect of prescription length. One notable exception was opioid prescribing, in which shorter initial prescription durations were consistently associated with lower risks of persistent opioid use and overdose. This may be because shorter prescriptions reduce the amount of opioids available for use and necessitate more frequent clinical follow-up, allowing healthcare providers to reassess treatment and minimize unnecessary continuation of opioid therapy. These findings suggest that the optimal prescription duration may differ according to medication type and clinical context. For example, longer prescription durations generally benefited patients receiving stable long-term therapies by improving medication adherence and reducing healthcare encounters, whereas shorter initial prescriptions appeared more appropriate for opioid therapy because of the increased risks of persistent use and overdose associated with longer prescriptions. These findings reinforce that prescription-duration decisions should be individualized according to the therapeutic indication and treatment objectives. Despite the limited evidence, these outcomes collectively provide a useful evaluation framework for assessing the broader impact of prescription-length policies. The findings of this review suggest that prescription duration should be tailored to the medication type, patient needs, treatment stability, and clinical context. Healthcare providers and policymakers should consider these factors when determining the most appropriate prescription duration. Although medication adherence and medication waste have been well studied, evidence for several other outcome domains, including clinical outcomes, healthcare utilization, retention in care, and patient satisfaction, remains limited. In addition, substantial methodological heterogeneity across existing studies, including differences in study designs, outcome definitions, measurement methods, and analytical perspectives, limits direct comparison of findings. Future research should therefore focus on addressing these evidence gaps and methodological challenges rather than simply increasing the number of studies.

Substantial methodological variation was observed across studies evaluating prescription-length policies. Differences in study design, outcome definitions, follow-up duration, and approaches to economic evaluation limited direct comparison of findings across studies. For example, medication adherence was assessed using multiple measures, including MPR, proportion of days covered (PDC), and self-reported adherence, while medication waste, healthcare utilization, and economic outcomes were measured using different definitions and analytical approaches. These methodological differences likely contributed to the heterogeneity in findings and made comparisons across studies more challenging. While prescription-length evaluations should remain tailored to different medications and clinical settings, more consistent outcome definitions, transparent reporting, and clearly defined analytical perspectives would improve the interpretation and comparison of findings within comparable clinical contexts. Such consistency would also facilitate more robust evidence synthesis where studies are sufficiently comparable, while preserving the flexibility needed to evaluate prescription-length practices across different medications and healthcare settings.

Collectively, the findings of this review suggest that prescription-length policies should be regarded as health system interventions rather than purely administrative decisions. Although prescription length is often viewed as an operational aspect of healthcare delivery, this review demonstrates that it can influence patient outcomes, healthcare utilization, and resource allocation across the healthcare system. These findings indicate that prescription-length policies should not adopt a one-size-fits-all approach but should instead consider medication characteristics, the likelihood of treatment modification, and the clinical context. However, these implications should be interpreted in the context of the available evidence, which was derived predominantly from studies conducted in high-income countries with formally regulated prescription-length policies. Therefore, the applicability of these findings to healthcare systems with different prescribing and medication access models should be considered with caution. In addition, implementation should be accompanied by ongoing monitoring and evaluation to assess both intended and unintended consequences across stakeholders, including patients, healthcare providers, payers, and community pharmacies. For instance, Murphy et al. (2012) found that 90-day prescriptions filled at retail pharmacies resulted in slightly less medication waste than those filled via mail-order, though the difference was minimal.^[13]^ To mitigate this waste when expanding long-duration prescriptions, systems should introduce supportive strategies like pharmacist-led medication reviews, refill synchronization, and dose-adjustment monitoring. Ultimately, these targeted strategies can ensure prescription-length policies succeed, although their effectiveness should be evaluated within different healthcare system contexts.

This review provides a comprehensive synthesis of the impact of prescription length across multiple clinical, economic, and healthcare system outcomes, while also identifying important methodological considerations for future research. By integrating evidence across these outcome domains, this review highlights the broader clinical, economic, and healthcare system implications of prescription-length policies. Several limitations should, however, be considered when interpreting the findings. Most included studies were observational, limiting causal inference. Considerable heterogeneity in study populations, medications, outcome definitions, follow-up periods, and analytical methods limited direct comparison across studies. Meta-analysis was, therefore, not feasible to draw conclusive numerical impacts of varying prescription lengths on particular outcomes. In addition, evidence for several outcome domains, particularly clinical outcomes, healthcare utilization, retention in care, and patient satisfaction, was limited, reducing the certainty of conclusions for these outcomes. In addition, most included studies were conducted in high-income countries with formally regulated prescription-length policies. Therefore, the generalizability of these findings to low- and middle-income countries, where medication access may not always be governed by formal prescription requirements, should be interpreted with caution.

## 5 Conclusion

Overall, the available evidence suggests that longer prescription durations were generally associated with improved medication adherence but also increased medication waste, whereas evidence for other economic, clinical and healthcare outcomes remained limited or heterogeneous. The impact of prescription length varied according to medication type, clinical context and treatment objectives, indicating that no single prescription duration is likely to be appropriate across all patient populations and therapeutic areas. Healthcare providers and policymakers should consider medication type, patient needs, treatment stability and healthcare system context when determining prescription duration. Because evidence for several outcomes was limited and study designs and outcome measures varied considerably across studies, these findings should be interpreted within the context of the available evidence. Further research using robust study designs and standardized outcome reporting would strengthen the evidence base for prescription-length policies.

## Declaration of generative AI use

The authors used ChatGPT (OpenAI) and Gemini (Google) to improve the language of this manuscript. The authors reviewed and edited the final version and take full responsibility for its content.

## CRediT authorship contribution statement

**Su Myat Thin:** Writing – original draft, Visualization, Validation, Software, Formal analysis, Data curation, Conceptualization. **Parnnaphat Luksameesate:** Writing – review & editing, Visualization, Methodology, Conceptualization. **Chanthawat Patikorn:** Writing – review & editing, Visualization, Validation, Methodology, Conceptualization. **Suthira Taychakhoonavudh:** Writing – review & editing, Visualization, Supervision, Project administration, Funding acquisition, Formal analysis, Conceptualization.

## Ethical issues

This study is the systematic review using the published articles found in the databases. Therefore, the ethical issue is not needed for this work.

## Funding

The first author was support by the Second Century Fund (C2F), 10.13039/501100002873Chulalongkorn University (Grant No. 10/2025), Thailand, through a postdoctoral fellowship. The funder had no role in the design, conduct, analysis, interpretation, or reporting of the study.

## Declaration of competing interest

The authors declare that they have no known competing financial interests or personal relationships that could have appeared to influence the work reported in this paper.
