# Supplementary material for: Medication and health-related outcomes by prescription lengths: A systematic review
Source: Explor Res Clin Soc Pharm. 2026 Oct 14;24:100862. doi: 10.1016/j.rcsop.2026.100862 (PMC13717161; doi:10.1016/j.rcsop.2026.100862)
Supplement: Supplementary file 1 [file mmc1.docx]

Medication and Health-Related Outcomes by Prescription Lengths: A Systematic Review of Global Evidence

Appendix A: Searching strategy

| Database | Search Date | Keywords | No. of studies |
| --- | --- | --- | --- |
| PubMed | 1.07.2026 | (((((((("prescription length") OR ("prescription duration")) OR ("medication duration")) OR ("prescription period")) OR ("extended prescription")) OR ("dispensing duration")) OR ("prescription size")) OR ("prescription interval")) OR ("prescription pattern")  Filter- English, Humans, from 2000 – 2025; | 860 |
| Scopus | 1.07.2026 | “prescription length” OR “prescription duration” OR “medication duration” OR “prescription period” OR “extended prescription” OR “dispensing duration” OR “prescription size” OR “prescription interval”  Filter- Document type: Article; Year: 2000-2025; Language: English; | 640 |
| ScienceDirect | 1.07.2026 | “prescription length” OR “prescription duration” OR “medication duration” OR “prescription period” OR “extended prescription” OR “dispensing duration” OR “prescription size” OR “prescription interval”  Filter- Title, abstract, keywords; Year: 2000-2025; Article type: Research articles; Languages: English; | 198 |
| Total | | | 1,698 |

Appendix B: Quality assessment scoring for included studies

Newcastle–Ottawa Quality Assessment of the Non-randomized studies

| Study | Selection | | | | Comparability | | Outcome | | | Overall quality |
| --- | --- | --- | --- | --- | --- | --- | --- | --- | --- | --- |
|  | 1 | 2 | 3 | 4 | 5 | 6 | 7 | 9 | 10 |  |
| Murphy et al., 2012 | * | * | * | * | * | * | * | * | * | High |
| Taitel et al., 2012 | * | * | * | * | * | * | * | * | * | High |
| Rabbani et al., 2009 | * | * | * | * | * | * | * | * |  | High |
| Walton, 2001 | * | * | * | * | * |  | * | * | * | High |
| Batal et al., 2007 | * | * | * | * | * | * | * | * | * | High |
| Doble et al., 2017 | * | * | * | * | * | * | * | * | * | High |
| Schetman et al., 2002 | * | * | * | * | * | * | * | * | * | High |
| Jin et al., 2020 | * | * | * | * | * |  | * | * | * | High |
| Parrish et al., 2021 | * | * | * | * | * | * | * | * | * | High |
| Pfeiffer et al., 2012 | * | * | * | * | * | * | * | * | * | High |
| Wen et al., 2022 | * | * | * | * | * | * | * | * | * | High |
| Campbell et al., 2021 | * | * | * | * | * | * | * | * | * | High |
| Gomes et al., 2021 | * | * | * | * | * | * | * | * | * | High |
| Zin et al., 2019 | * | * | * | * | * |  | * | * | * | High |

Note: * =reported item in the study

JBI Critical Appraisal Checklist for Quasi-Experimental Studies

| Items | Jarujumrus et al., 2023 | Hermes et al., 2010 | Amin et al., 2017 | Domino et al., 2011 |
| --- | --- | --- | --- | --- |
| Is it clear in the study what is the cause and what is the effect? | Yes | Yes | Yes | Yes |
| Were the participants included in any comparisons similar? | Yes | Yes | Yes | Yes |
| Were the participants receiving similar treatment/care other than the intervention? | Yes | Yes | Yes | Yes |
| Was there a control group? | Yes | Yes | Yes | Yes |
| Were there multiple measurements of the outcome both pre and post intervention? | No | Yes | Yes | Yes |
| Was follow-up complete and adequately described/analyzed? | Yes | Yes | Yes | Yes |
| Were outcomes measured in the same way for all participants? | Yes | Yes | Yes | Yes |
| Were outcomes measured in a reliable way? | Yes | Yes | Yes | Yes |
| Was appropriate statistical analysis used? | Yes | Yes | Yes | Yes |
| Overall quality | High | High | High | High |

ECOBIAS Checklist

|  | Items | Gaziano et al., 2015 | Domino et al., 2004 |
| --- | --- | --- | --- |
| Overall checklist for bias in economic evaluation | Narrow perspective bias | No | Yes |
|  | Inefficient comparator bias | No | No |
|  | Cost measurement omission bias | Partly | Partly |
|  | Intermittent data collection bias | NA | NA |
|  | Invalid valuation bias | No | No |
|  | Ordinal ICER bias | Unclear | NA |
|  | Double-counting bias | No | No |
|  | Inappropriate discounting bias | No | NA |
|  | Limited sensitivity analysis bias | No | Yes |
|  | Sponsor bias | Unclear | Unclear |
|  | Reporting and dissemination bias | No | No |
| Model-specific aspects of bias in economic evaluation | Structural assumptions bias | No | NA |
|  | No treatment comparator bias* | No | NA |
|  | Wrong model bias | No | NA |
|  | Limited time horizon bias | No | No |
|  | Bias related to data identification | Partly | Yes |
|  | Bias related to baseline data | No | Yes |
|  | Bias related to treatment effects | Partly | NA |
|  | Bias related to quality-of-life weights (utilities) | No | NA |
|  | Non-transparent data incorporation bias | No | Yes |
|  | Limited scope bias | No | No |
|  | Bias related to internal consistency | Unclear | Unclear |
|  | Overall quality | Good | Moderate |

Note: NA = Not Applicable,

Mixed Methods Appraisal Tool (MMAT) 2018/2024

| Items | Ntjikelane et al., 2025 |
| --- | --- |
| Are there clear research questions? | Yes |
| Do the collected data allow to address the research questions? | Yes |
| Is there an adequate rationale for using a mixed methods design to address the research question? | Yes |
| Are the different components of the study effectively integrated to answer the research question? | Yes |
| Are the outputs of the integration of qualitative and quantitative components adequately interpreted? | Yes |
| Are divergences and inconsistencies between quantitative and qualitative results adequately addressed? | Not reported |
| Do the different components of the study adhere to the quality criteria of each tradition of the methods involved? | Yes |
| Overall quality | High |

JBI Critical Appraisal Checklist for Analytical Cross-Sectional Studies

| Items | Schmittdiel et al., 2015 |
| --- | --- |
| Were the criteria for inclusion in the sample clearly defined? | Yes |
| Were the study subjects and the setting described in detail? | Yes |
| Was the exposure measured in a valid and reliable way? | Yes |
| Were objective, standard criteria used for measurement of the condition? | Yes |
| Were confounding factors identified? | Yes |
| Were strategies to deal with confounding factors stated? | Yes |
| Were the outcomes measured in a valid and reliable way? | Yes |
| Was appropriate statistical analysis used? | Yes |
| Overall quality | High |

JBI Critical Appraisal Checklist for Qualitative Studies

| Items | Wilson et al., 2013 |
| --- | --- |
| Is there congruity between the stated philosophical perspective and the research methodology? | Yes |
| Is there congruity between the research methodology and the research question or objectives? | Yes |
| Is there congruity between the research methodology and the methods used to collect data? | Yes |
| Is there congruity between the research methodology and the representation and analysis of data? | Yes |
| Is there congruity between the research methodology and the interpretation of results? | Yes |
| Is there a statement locating the researcher culturally or theoretically? | No |
| Is the influence of the researcher on the research, and vice-versa, addressed? | No |
| Are participants, and their voices, adequately represented? | Yes |
| Is the research ethical according to current criteria or, for recent studies, is there evidence of ethical approval by an appropriate body? | Yes |
| Do the conclusions drawn in the research report flow from the analysis, or interpretation, of the data? | Yes |
| Overall quality | High |

Appendix C: Information of excluded studies

| Title of studies (ref.) | Reason of exclusion |
| --- | --- |
| The Effect of Prescription Size on Acquisition of Maintenance Medications (Steiner et al., 1993) | Not reported about outcome data of prescription lengths |
| Prescription patterns of quetiapine for multiple drug abuse, depression, and psychosis- A retrospective study (Al-Qaaneh et al., 2023) | No specific data for prescription length |
| Antimicrobial prescribing after rapid influenza PCR implementation in the emergency department (Wesolowski et al., 2023) | No specific data for prescription length |
| Cost of Drug Wastage from Dose Modification and Discontinuation of Oral Anticancer Drugs (Lam et al., 2023) | No specific data for prescription length changes |
| Ninety-day versus thirty-day drug-dispensing systems (Parikh et al., 2001) | No specific data for prescription length changes |
| Does 90-day prescription supply at retail improve compliance in a managed care setting? (Thiebaud et al., 2006) | No specific data for prescription length changes |
| Opioid Prescribing Patterns After Imposition of Setting-Specific Limits on Prescription Duration (Allen et al., 2024) | No specific data for prescription length changes |
| Initial proton pump inhibitor characteristics associated with long-term prescriptions in US veterans diagnosed with gastro-oesophageal reflux disease (Gawron et al., 2014) | Not reported about outcome data of prescription lengths |
| Wasted Medication - How Big Is The Problem? (Ryvkin et al., 2009) | Not full text |
| Comparing Medication Adherence And Wastage Among Three Different Retail Programs (Jiang et al., 2007) | Not full text |

Table S1: Characteristics of the included studies

| Author, year  (study location) | Study population | Study design | No. of participants | Age (year)  [mean (SD)] | Focused disease | Medication length |
| --- | --- | --- | --- | --- | --- | --- |
| Chronic disease medications | | | | | | |
| Murphy et al., 2012 (USA) | Commercially insured adults using maintenance medications | Retrospective cohort | 60,358  (Retail-30: 35,418; Retail-90: 17,897; Mail: 6,033) | Retail-30: 51.2 (±12.2);  Retail-90: 56.3 (±11.1);  Mail: 58.4 (±11.3) | Diabetes, thyroid disorders, hypertension, dyslipidemia, & depression | 30 days Vs 90 days |
| Taitel et al., 2012 (USA) | Medicaid beneficiaries aged 18–64 using maintenance medications in 7 therapeutic classes | Retrospective cohort | 325,539 | Average-  30-day group: 42-55  90-day group: 51-60 | Hypertension, diabetes, dyslipidemia, depression, asthma/COPD, thyroid disorder, and GERD | 30 days Vs 90 days |
| Walton, 2001 (USA) | Veterans receiving simvastatin or lovastatin through outpatient services | Retrospective cohort | 16,990 prescriptions | NA | Hyperlipidemia | 30 days Vs 90 days |
| Rabbani et al., 2009 (USA) | Adults with chronic conditions (data from commercial insurance) | Retrospective cohort | 2,971 participants with 26,852 prescriptions | NA | Chronic diseases | 30 days Vs 90 days |
| Batal et al., 2007 (USA) | Patients receiving care and medications at Denver Health Medical Center (DHMC) over a three-year period. | Retrospective cohort | 3,386 | 57.8 (±10.9) | Lowering cholesterol level | 30 days Vs 60 days |
| Schetman et al., 2002 (USA) | Patients from a rural health center in Virginia with at least 2 prescriptions and 1-year of data | Retrospective cohort | 1,906 | 58.9 (±12.1) | Various chronic diseases | 30 days increment (max. 90 days) |
| Doble et al., 2017 (UK) | All adult patients (≥18 years old) receiving one or more prescriptions for at least one medication relevant to a case study of interest between 1 January 2004 and 31 December 2014 | Retrospective cohort | 50,000 | ≥18 | Type 2 diabetes mellitus, Myocardial infarction, & Depression | <60 days Vs ≥60 days |
| Jin et al., 2020 (China) | Patients with allergic rhinitis (AR) who started Sublingual immunotherapy (SLIT) between January 2017 and June 2018 | Retrospective cohort | 306 | 30.54 (±11.71) | Allergic rhinitis | <3 months Vs 3-6 months Vs <6 months |
| Pfeiffer et al., 2012 (USA) | Veterans with a new diagnosis of depression and a new antidepressant prescription | Retrospective cohort | 383,634 | <35 - 80+ | Depression | <90 days vs 90 days |
| Hermes et al., 2010 (USA) | Commercially insured members in January 2007 | Quasi-experimental | 183,666 | >18 | Chronic diseases (high cholesterol, hypertension, diabetes) | 30 days Vs 90 days |
| Jarujumrus et al., 2023 (Thailand) | Patients who received prescription lengths of 30 and 90 days based on information recorded in the hospital database between 2014 and 2017 | Quasi-experimental | 2,046 | >18 | Dyslipidemia & type-2 diabetes mellitus | 30 days Vs 90 days |
| Amin et al., 2017 (USA) | Medicaid enrollees taking medications for at least 1 of 3 specific medication categories for chronic conditions | Quasi-experimental | 162,248 with 252,869-921,069 observations | 18 - 64 | Hypertension, hyperlipidemia & psychotics disease | 100 days & <40 days |
| Domino et al., 2011 (USA) | North Carolina Medicaid enrollees affected by 2001 policy changes on days’ supply and copayments | Quasi-experimental | >260,000 prescriptions | NA | All conditions requiring maintenance medications | 100 days & 34 days |
| Domino et al., 2004 (USA) | Medicaid patients filling prescriptions in 6 chronic medication categories | Simulation | 255,000–783,000 prescription | NA | Chronic diseases | 100 days & 34 days |
| Gaziano et al., 2015 (South Africa) | Patients taking statins with prescriptions for longer time periods than the current norm | Simulation | 1,214 | ≥35 | High cholesterol & cardiovascular disease | 30 days Vs 60 days Vs 90 days |
| Schmittdiel et al., 2015 (USA) | Medicare-aged diabetes patients (≥65 years) | Cross-sectional | 129,040 | ≥65 | Type 2 diabetes, hypertension, and hyperlipidemia | 30 days Vs 31-60 days Vs 61-90 days Vs >90 days |
| Wilson et al., 2013 (UK) | Patients and carers with chronic conditions (e.g. diabetes, Parkinson’s, COPD), receiving community-based care services. | Cross-sectional | 30 | 39–92 | Chronic diseases | Repeated 28 days |
| Antiretroviral therapy (ART) | | | | | | |
| Parrish et al., 2021 (Haiti) | ART-naïve HIV-positive individuals enrolled in the clinics, who started ART on or after January 1, 2017, up until November 1, 2018, | Retrospective cohort | 21,880 | <15 - >55 | Acquired  immunodeficiency syndrome (AIDS) | <1 month to >5 months |
| Ntjikelane et al., 2025 (South Africa) | Healthcare providers (facility managers, nurses and pharmacists, and pharmacist assistants) at 24 primary healthcare clinics in South Africa from May to September 2024. | Mixed method | 149 | 37 - 53 | AIDS | 1-2 months Vs 3 months |
| Varenicline | | | | | | |
| Wen et al., 2022 (Taiwan) | Adult Taiwanese participants received varenicline monotherapy for smoking cessation between March 2012 and September 2016 | Retrospective cohort | 5,463 | 18 - 65 | Smoking cessation | 1 week Vs 2 weeks Vs ≥ 3weeks |
| Opioids | | | | | | |
| Campbell et al., 2021 (USA) | Opioid-naïve patients aged ≥13 years undergoing dental procedures, received opioid prescriptions, and had 180 days of continuous enrollment post-procedure. | Retrospective cohort | 786,125 | 39.9 (±19.6) | Post-dental procedure pain | 1−3 days Vs 4−7 days Vs 8−14 days Vs ≥15 days |
| Gomes et al., 2021 (Canada) | Ontario residents newly initiating opioids for pain between July 1, 2013, and March 31, 2016 | Retrospective cohort | 2,021,371 | >15 | Pain management | 1-2 days Vs 3-4 days Vs 5-7 days Vs 8-10 days Vs 11-29 days Vs 30+days |
| Zin et al., 2019 (Malaysia) | Patients had no opioid prescriptions in the 365 days prior between 1 January 2011 and 31 December 2016 | Retrospective cohort | 33,752 | ≧18 | Pain management | 3 days Vs 3-7 days Vs >7 days |

Note: NA= Not Applicable, SD= standard deviation, COPD= Chronic Obstructive Pulmonary Disease,

Table S2: Variations in findings by study design and outcome measurement across included studies

| Study design | Outcome measurement indicator | Prescription lengths (ref.) | Outcome measurement calculation or definition |
| --- | --- | --- | --- |
| Medication adherence | | | |
| Retrospective cohort | MPR | 30D Vs 90D (Taitel et al., 2012) | MPR= sum of Days’ supply for each therapeutic/ Total number of days in the follow-up period (Taitel et al., 2012) |
|  | % of patients with adherence ≥80% | 30D Vs 60D (Batal et al., 2007) | Adherence score = Total days of medication obtained/ Total days in the study period  (High adherence= adherence score ≥80%) (Batal et al., 2007) |
|  | % of adherent patients | <3M Vs 3-6M Vs >6M (Jin et al., 2020)  30D increment (max. 90D) (Schectman et al., 2002) | Adherent patients = Patients who continued the treatment, terminated the treatment with the approval of a specialist, or stopped the treatment for less than 1 week and continued the treatment later (Jin et al., 2020)  Non-adherent patients = Patients who terminated the treatment for more than 1week without the approval of a specialist (Jin et al., 2020)  Adherence = Total days of therapy dispensed (excluding the last refill)/ Number of days between the first and last refill (Schectman et al., 2002) |
|  | % of patients refilling their medications | <90D vs 90D (Pfeiffer et al., 2012) | Refilling the medication at least once within the 120-day period following the index prescription. (Pfeiffer et al., 2012) |
| Quasi-experimental | % of adherent patients with PDC ≥80% | 30D Vs 90D (Hermes et al., 2010) | Adherence = the proportion of days covered (PDC) and an average PDC ≥ 80% (Hermes et al., 2010) |
|  |  | 100D to <40 D (Amin et al., 2017, Domino et al., 2011) | PDC = Number of days covered by medication / Total number of days in the observation period (Amin et al., 2017, Domino et al., 2011) (Adherent if PDC ≥80%) |
|  | % of adherence with MPR ≥80% | 30D to 90D (Jarujumrus et al., 2023) | MPR = Total days of medication provided / Total days of follow – up (Jarujumrus et al., 2023) |
| Cross-sectional | Adherence with PDC ≥80% | 30D Vs 31D-60D Vs 61D-90D Vs >90D (Schmittdiel et al., 2015) | PDC =the percent of days in the measurement period “covered” by prescription fills for the same medication or medications in the same therapeutic category. (Adherent if PDC ≥80%) (Schmittdiel et al., 2015) |
|  | Medication adherence (qualitative) | 1-2M Vs 3M (Ntjikelane et al., 2025) | Qualitative question: Enhancing medication adherence. (Ntjikelane et al., 2025) |
| Medication waste | | | |
| Retrospective cohort | Medication day supply waste | 30D Vs 90D (Taitel et al., 2012, Murphy et al., 2012, Walton, 2001) | Medication waste = the sum of the excess days-supply/ the total number of fills which were converted to 30-day equivalents (Murphy et al., 2012)  Average wastage days (mean days of wastage) = sum of days of wastage/ total number of patients (Taitel et al., 2012)  (Medication waste = a switch of drug type or strength within the same therapeutic class that occurred before the expected refill date) (Taitel et al., 2012)  The mean quantity (day) of drug wasted in each group = the average quantity dispensed - the average quantity used for each group. (Walton, 2001) |
|  |  | <60D Vs ≥60D (Doble et al., 2017) | Medication wastage = mean number of days’ supply wasted days for two prescription length groups over the 11-year period (Doble et al., 2017) |
| Cost perspective outcomes | | | |
| Waste cost | | | |
| Retrospective cohort | Cost difference ($) | 30D Vs 90D (Taitel et al., 2012) | Waste cost = the wastage days x the average daily cost of the 90-day group (Taitel et al., 2012) |
|  | Mean cost (£) | <60D Vs ≥60D (Doble et al., 2017) | Medication wastage cost = Mean cost of wastage per prescription (Doble et al., 2017) |
| Costs of target medications | | | |
| Quasi-experimental | Cost difference ($) | 100D 🡪 34D (Domino et al., 2011) | Costs of target medications = expenditures that individuals had average quarterly spending on target medications recorded in the Medicaid claims files (Domino et al., 2011) |
| Out-of-pocket cost | | | |
| Retrospective cohort | Mean cost ($) | 30D Vs 90D (Rabbani et al., 2009) | Out-of-pocket cost = drug cost paid by patient (Rabbani et al., 2009) |
| Dispensing fee-related cost | | | |
| Retrospective cohort | Cost ($) | 30D Vs 90D (Walton, 2001) | Dispensing fee-related cost = unit cost of dispensing fee that contributes to the total unnecessary cost per a prescription over a year (Walton, 2001) |
|  | Mean cost (£) | <60D Vs ≥60D (Doble et al., 2017) | Dispensing fee-related cost = mean cost of dispensing fee charged by pharmacists, contributing to the total unnecessary cost per a prescription in the NHS (Doble et al., 2017) |
| Simulation | Cost ($) | 100D 🡪 34D (Domino et al., 2004) | Dispensing fee-related cost = additional cost paid to the pharmacy for each prescription fill following the policy change from a 100-day to 34-day supply within a fiscal year. (Domino et al., 2004) |
| GP time cost | | | |
| Retrospective cohort | Mean cost (£) | <60D Vs ≥60D (Doble et al., 2017) | GP time cost = the cost related to the time of a general practitioner (GP) for producing a prescription (Doble et al., 2017) |
| Pharmacist labor time cost | | | |
| Simulation | Cost difference ($) | 30D Vs 60D Vs 90D (Gaziano et al., 2015) | Pharmacist labor time cost = the cost related to pharmacist service for a prescription (Gaziano et al., 2015) |
| Patient time cost | | | |
| Simulation | Cost difference ($) | 30D Vs 60D Vs 90D (Gaziano et al., 2015) | Patient time cost = the cost related to patient’s time for traveling to and from the pharmacy (Gaziano et al., 2015) |
| Transportation cost | | | |
| Simulation | Cost difference ($) | 30D Vs 60D Vs 90D (Gaziano et al., 2015) | Transportation cost = the travel cost for a patient to travel to and from the pharmacy (Gaziano et al., 2015) |
| Total unnecessary cost | | | |
| Retrospective cohort | Cost ($) | 30D Vs 90D (Walton, 2001) | Total unnecessary cost = [(daily cost of the drug x quantity of drug wasted)+ dispensing cost] x (each set period of days/ number of days used) - dispensing cost (Walton, 2001) |
|  | Cost (£) | <60D Vs ≥60D (Doble et al., 2017) | Total unnecessary cost = (cost of wastage + cost of dispensing fee+ cost of prescriber times) x (120/  Mean days used) – (cost of dispensing fee+ cost of prescriber times) (Doble et al., 2017) |
| Net cost impact | | | |
| Retrospective cohort | Cost difference ($) | 30D Vs 90D (Taitel et al., 2012) | Gross Savings= (Cost per day in 30 days −Cost per day in 90 days) x Total days’ supply in 30-day group (Taitel et al., 2012) |
| Simulation | Cost difference ($) | 30D Vs 60D Vs 90D (Gaziano et al., 2015) | Cost saving = Total cost of 30-day group – Total cost of other longer prescription length group (Gaziano et al., 2015)  (Total cost of each group = transportation cost + patient direct time cost + pharmacist labor cost + medication cost + cardiovascular disease event cost) |
| Total expenditure | | | |
| Retrospective cohort | Mean cost ($) | 30D Vs 90D (Rabbani et al., 2009) | Total expenditure (monthly) = monthly out-of-pocket for drugs paid by patient + monthly medication expenditure paid by third-party payers (Rabbani et al., 2009) |
| Quasi-experimental | Cost difference ($) | 100D 🡪 34D (Domino et al., 2011) | Total expenditure = all expenditures for each enrollee per quarter, on services, pharmaceuticals, and other items recorded in the Medicaid claims data (Domino et al., 2011) |
| Adverse medication use | | | |
| Overdose | | | |
| Retrospective cohort | Risk of opioid overdose | 1-2D Vs 3-4D Vs 5-7D Vs 8-10D Vs 11-29D Vs 30+D (Gomes et al., 2021) | Overdose = event of opioid-related overdose risk during follow-up (Gomes et al., 2021) |
| Persistent use | | | |
| Retrospective cohort | Persistent opioid use | 1−3D Vs 4−7D Vs 8−14D Vs ≥15D (Campbell et al., 2021) | Persistent opioid use = ≥1 opioid prescription within 90 days & ≥1 opioid prescription during 90- 365 days after initiation (Campbell et al., 2021) |
|  | Risk of ongoing opioid use | 1-2D vs 3-4D vs 5-7D vs 8-10D vs 11-29D vs 30+D (Gomes et al., 2021) | Persistent use = event of ongoing opioid use at 1 year following the index prescription (Gomes et al., 2021) |
|  | Long-term opioid use | 3-7D vs >7D (Zin et al., 2019) | Persistent use = receiving opioid prescriptions for ≥90 days within the 12 months after the initial prescription (Zin et al., 2019) |
| Clinical outcome | | | |
| Retrospective cohort | Final serum cholesterol value | 30D Vs 60D (Batal et al., 2007) | Health outcome = measuring the patients’ final serum cholesterol levels in 30-day and 60-day groups (Batal et al., 2007) |
|  | Immediate or complete quitting smoking | 1 W Vs 2 W Vs ≥ 3W (Wen et al., 2022) | Quitting smoking outcome = smoking cessation status based on self-reported cessation status obtained from the 6-month follow-up survey (Wen et al., 2022) |
| Simulation | Lifetime events averted | 30D Vs 60D Vs 90D (Gaziano et al., 2015) | Lifetime events averted = reduction in the number of fatal and nonfatal cardiovascular-disease events over the lifetime of the simulated population in each longer prescription length group compared to 30-day prescription length group. (Gaziano et al., 2015) |
| Healthcare utilization | | | |
| Retrospective cohort | Healthcare encounter | <90D vs 90D (Pfeiffer et al., 2012) | Healthcare encounter = percentage of patients for clinical encounter during the 90 days before and after the index diagnosis (Pfeiffer et al., 2012) |
| Mixed method | Patient clinic visit | 1-2M Vs 3M (Ntjikelane et al., 2025) | Retention in care = percentage of healthcare providers answering 3-month supply reducing clinic visits of patients comparing to 1-2 months (Ntjikelane et al., 2025) |
| Retention in care | | | |
| Retrospective cohort | Retention in care | <1M to >5M (Parrish et al., 2021) | Retention in care or timely ART pickup = picking up ART refill within 30 days of scheduled pickup at 12 months (Parrish et al., 2021) |
| Patient satisfaction | | | |
| Mixed method | Convenience for patients | 1-2M Vs 3M (Ntjikelane et al., 2025) | Patient convenience = percentage of healthcare providers answering patients’ convenience in 3-month and 1–2-month groups (Ntjikelane et al., 2025) |
| Cross-sectional qualitative | Patient satisfaction | Repeated 28D (Wilson et al., 2013) | Patient satisfaction = qualitative answer by patients about their satisfaction for 28 or 30-day prescription length (Wilson et al., 2013) |

Note: D= days, M= months, W= weeks, MPR= medication possession ratio, PDC= proportion of days covered, ART= antiretroviral therapy,

Table S3: Adverse medication outcomes

| Study design | Study, country  (Prescription length) | Medication | Overdose | Persistent use |
| --- | --- | --- | --- | --- |
| Retrospective cohort | Campbell et al., 2021, USA (1−3D Vs 4−7D Vs 8−14D Vs ≥15D) | Opioids |  | 1-3D= 1 (reference group, AOR)  4-7D= 1.02 (0.99, 1.04) ^n^  8-14D= 1.22 (1.15, 1.30) *  >15D= 2.63 (2.37, 2.91) *  long-acting opioid = 7.88 (6.29, 9.87)* |
|  | Gomes et al., 2021, Canada (1-2D vs 3-4D vs 5-7D vs 8-10D vs 11-29D vs 30+D) |  | 1-2D= 1 (reference group, Hazard ratio)  3-4D= 1.07 (95% CI: 0.89 to 1.29) ^n^  5-7D= 1.27 (95% CI: 1.05 to 1.55)*  8-10D= 1.51 (95% CI: 1.18 to 1.94)*  11-29D= 1.80 (95% CI: 1.36 to 2.38)*  30+D= 1.71 (95% CI: 1.21 to 2.41)* | 1-2D= 1 (reference group, AOR)  3-4D= 1.19 (1.15 to 1.24)*  5-7D= 1.95 (1.88 to 2.02)*  8-10D= 2.77 (2.66 to 2.88)*  11-29D= 4.62 (4.45 to 4.81)*  30+D= 8.02 (7.69 to 8.36)* |
|  | Zin et al., 2019, Malaysia  (3-7D vs >7D) |  |  | <3D= 1 (reference group, AOR)  3-7D= 1.06 ^n^  >7D= 16.47* |

Note: D= days, * = significant, n = non-significant, AOR= adjusted odds ratio, Yellow color = harmful impact on outcomes while changing prescription lengths,

Table S4: Clinical outcomes

| Study design | Study, country  [Prescription length]  (outcome measure) | Medication | Clinical outcome |
| --- | --- | --- | --- |
| Retrospective cohort | Batal et al., 2007, USA  [30D Vs 60D] (Final serum cholesterol value) | Statins | 30D = 191.5 ± 52.6 mg/dL (4.96 ±1.36 mmol/L)*  60D = 185.3 ± 46.2 mg/dL (4.80 ±  1.20 mmol/L)* |
| Simulation | Gaziano et al., 2015, South Africa  [30D Vs 60D Vs 90D] (lifesaving events) |  | 30D to 60D: 1694 ^a^ 30D to 90D: 2553 ^a^ |
| Retrospective cohort | Wen et al., 2022, Taiwan  [1 W Vs 2 W Vs ≥ 3W] (immediate/complete quitting Vs non-quitting) | Varenicline | OR= 1.19 (1.15, 1.22) * |

Note: D= days, W= weeks * = significant, a = no data about significance of the result, n = non-significant, OR= odds ratio, Green color = beneficial impact on outcomes while changing prescription lengths,

Table S5: Healthcare utilization, retention in care and satisfaction of patients

| Study design | Study, country  (Prescription length) | Medication | Healthcare utilization | Retention in care | Patient satisfaction |
| --- | --- | --- | --- | --- | --- |
| Retrospective cohort | Parrish et al., 2021, Haiti  (<1 month to >5 months) | ART |  | 30D= 24.2%-point gain (95%CI: 21.9, 26.5) 110D= 5.1%-points (95%CI: 2.4,7.8) |  |
| Mixed method | Ntjikelane et al., 2025, South Africa  (1-2months Vs 3 months) |  | Patient clinic visit: 87% of HCP answered 3-month supply reducing clinic visits comparing to 1-2months |  | 76% of HCP answered that 3-month supply was convenience for patients |
| Retrospective cohort | Pfeiffer et al., 2012, USA  (<90D vs 90D) | Antidepressive | Healthcare encounter:  <90D= 24.9% 90D= 13.7% |  |  |
| Cross-sectional qualitative | Wilson et al., 2013, UK  (repeated 28 D) | Multiple drugs |  |  | Patients were with less satisfaction for 28 or 30D |

Note: D= days, ART= antiretroviral therapy, UTI= urinary tract infection, HCP= healthcare providers, * = significant, Green color = beneficial impact on outcomes while changing prescription lengths, Yellow color = harmful impact on outcomes while changing prescription lengths,

Supplementary: PRIMSA Checklist

| **Section and Topic** | **Item #** | **Checklist item** | **Location where item is reported** |
| --- | --- | --- | --- |
| **TITLE** | | |  |
| Title | 1 | Identify the report as a systematic review. | Page 1 |
| **ABSTRACT** | | |  |
| Abstract | 2 | See the PRISMA 2020 for Abstracts checklist. | Page 1 |
| **INTRODUCTION** | | |  |
| Rationale | 3 | Describe the rationale for the review in the context of existing knowledge. | Page 2 |
| Objectives | 4 | Provide an explicit statement of the objective(s) or question(s) the review addresses. | Page 2 |
| **METHODS** | | |  |
| Eligibility criteria | 5 | Specify the inclusion and exclusion criteria for the review and how studies were grouped for the syntheses. | Page 2 |
| Information sources | 6 | Specify all databases, registers, websites, organisations, reference lists and other sources searched or consulted to identify studies. Specify the date when each source was last searched or consulted. | Page 2 |
| Search strategy | 7 | Present the full search strategies for all databases, registers and websites, including any filters and limits used. | Page 2 & supplementary |
| Selection process | 8 | Specify the methods used to decide whether a study met the inclusion criteria of the review, including how many reviewers screened each record and each report retrieved, whether they worked independently, and if applicable, details of automation tools used in the process. | Page 2 |
| Data collection process | 9 | Specify the methods used to collect data from reports, including how many reviewers collected data from each report, whether they worked independently, any processes for obtaining or confirming data from study investigators, and if applicable, details of automation tools used in the process. | Page 2 |
| Data items | 10a | List and define all outcomes for which data were sought. Specify whether all results that were compatible with each outcome domain in each study were sought (e.g. for all measures, time points, analyses), and if not, the methods used to decide which results to collect. | Page 2 |
|  | 10b | List and define all other variables for which data were sought (e.g. participant and intervention characteristics, funding sources). Describe any assumptions made about any missing or unclear information. | Not performed |
| Study risk of bias assessment | 11 | Specify the methods used to assess risk of bias in the included studies, including details of the tool(s) used, how many reviewers assessed each study and whether they worked independently, and if applicable, details of automation tools used in the process. | Page 2 & supplementary |
| Effect measures | 12 | Specify for each outcome the effect measure(s) (e.g. risk ratio, mean difference) used in the synthesis or presentation of results. | Page 2 |
| Synthesis methods | 13a | Describe the processes used to decide which studies were eligible for each synthesis (e.g. tabulating the study intervention characteristics and comparing against the planned groups for each synthesis (item #5)). | Page 2 |
|  | 13b | Describe any methods required to prepare the data for presentation or synthesis, such as handling of missing summary statistics, or data conversions. | Page 2 |
|  | 13c | Describe any methods used to tabulate or visually display results of individual studies and syntheses. | Page 2 |
|  | 13d | Describe any methods used to synthesize results and provide a rationale for the choice(s). If meta-analysis was performed, describe the model(s), method(s) to identify the presence and extent of statistical heterogeneity, and software package(s) used. | Page 2 |
|  | 13e | Describe any methods used to explore possible causes of heterogeneity among study results (e.g. subgroup analysis, meta-regression). | Not performed |
|  | 13f | Describe any sensitivity analyses conducted to assess robustness of the synthesized results. | Not performed |
| Reporting bias assessment | 14 | Describe any methods used to assess risk of bias due to missing results in a synthesis (arising from reporting biases). | Page 2 & supplementary |
| Certainty assessment | 15 | Describe any methods used to assess certainty (or confidence) in the body of evidence for an outcome. | Not performed |
| **RESULTS** | | |  |
| Study selection | 16a | Describe the results of the search and selection process, from the number of records identified in the search to the number of studies included in the review, ideally using a flow diagram. | Page 2 &3 |
|  | 16b | Cite studies that might appear to meet the inclusion criteria, but which were excluded, and explain why they were excluded. | Page 3 & supplementary |
| Study characteristics | 17 | Cite each included study and present its characteristics. | Page 3 |
| Risk of bias in studies | 18 | Present assessments of risk of bias for each included study. | Page 3 |
| Results of individual studies | 19 | For all outcomes, present, for each study: (a) summary statistics for each group (where appropriate) and (b) an effect estimate and its precision (e.g. confidence/credible interval), ideally using structured tables or plots. | Page 3 |
| Results of syntheses | 20a | For each synthesis, briefly summarise the characteristics and risk of bias among contributing studies. | Page 3 |
|  | 20b | Present results of all statistical syntheses conducted. If meta-analysis was done, present for each the summary estimate and its precision (e.g. confidence/credible interval) and measures of statistical heterogeneity. If comparing groups, describe the direction of the effect. | Page 3-5 |
|  | 20c | Present results of all investigations of possible causes of heterogeneity among study results. | Page 3-5 |
|  | 20d | Present results of all sensitivity analyses conducted to assess the robustness of the synthesized results. | - |
| Reporting biases | 21 | Present assessments of risk of bias due to missing results (arising from reporting biases) for each synthesis assessed. | Page 3 |
| Certainty of evidence | 22 | Present assessments of certainty (or confidence) in the body of evidence for each outcome assessed. | Not performed |
| **DISCUSSION** | | |  |
| Discussion | 23a | Provide a general interpretation of the results in the context of other evidence. | Page 5-7 |
|  | 23b | Discuss any limitations of the evidence included in the review. | Page 7 |
|  | 23c | Discuss any limitations of the review processes used. | Page 7 |
|  | 23d | Discuss implications of the results for practice, policy, and future research. | Page 7 |
| **OTHER INFORMATION** | | |  |
| Registration and protocol | 24a | Provide registration information for the review, including register name and registration number, or state that the review was not registered. | Not performed |
|  | 24b | Indicate where the review protocol can be accessed, or state that a protocol was not prepared. | Not performed |
|  | 24c | Describe and explain any amendments to information provided at registration or in the protocol. | Not performed |
| Support | 25 | Describe sources of financial or non-financial support for the review, and the role of the funders or sponsors in the review. | Page 7 |
| Competing interests | 26 | Declare any competing interests of review authors. | Page 7 |
| Availability of data, code and other materials | 27 | Report which of the following are publicly available and where they can be found: template data collection forms; data extracted from included studies; data used for all analyses; analytic code; any other materials used in the review. | Not performed |

*From:*  Page MJ, McKenzie JE, Bossuyt PM, Boutron I, Hoffmann TC, Mulrow CD, et al. The PRISMA 2020 statement: an updated guideline for reporting systematic reviews. BMJ 2021;372:n71. doi: 10.1136/bmj.n71. This work is licensed under CC BY 4.0. To view a copy of this license, visit <https://creativecommons.org/licenses/by/4.0/>
